# Altered Membrane Protein Abundance in the Cerebral Cortex of the JNPL3 Mouse Model of Tauopathy

**DOI:** 10.1021/acs.jproteome.6c00679

**Published:** 2026-09-16

**Authors:** Sabrina Petralla, Jooseppi Hyvärinen, Mikko Gynther, Alexey Afonin, Marko Lehtonen, Seppo Auriola, Maria Panayotova, Katja M. Kanninen, Gert Fricker, Elena Puris

**Affiliations:** † Institute of Pharmacy and Molecular Biotechnology, Ruprecht-Karls-University, Heidelberg DE-69120, Germany; ‡ A.I. Virtanen Institute for Molecular Sciences, 163043 University of Eastern Finland, Kuopio FI-70211, Finland; § School of Pharmacy, University of Eastern Finland, Kuopio FI-70211, Finland; ∥ Institute of Pharmacology, Faculty of Medicine, Heidelberg University, Heidelberg DE-69120, Germany

**Keywords:** tauopathy, membrane proteins, JNPL3 mice, ABC transporters, solute carrier, sex

## Abstract

Developing effective therapeutic approaches for tau-associated neurodegenerative diseases, including frontotemporal dementia and Alzheimer’s disease, remains extremely challenging due to their complex pathogeneses. Membrane proteins constitute a large proportion of current therapeutic and drug delivery targets because of their key roles in essential cellular processes. Here, we examined alterations in membrane protein abundances in the prefrontal cortex of male and female mouse models of human tauopathy, JNPL3 mice, compared with sex-matched wild-type (WT) mice using quantitative proteomics. The data-independent acquisition (DIA) proteomics analysis revealed various alterations in membrane proteins involved in different altered pathways in male JNPL3 mice (e.g., nucleotide metabolism and translation) and female JNPL3 mice (e.g., mitochondrial function). In addition, we found sex-associated changes in the abundances of several proteins previously reported to be involved in tau pathology, such as Reelin, DARPP-32, Zyxin, Tbce, and Gng5, in JNPL3 mice. Our multiple-reaction monitoring absolute quantitative analysis of transporter proteins revealed reduced abundance of the main drug efflux transporter ABCB1 in male JNPL3 mice compared with WT animals, with no changes in female groups. All in all, we provide novel information on membrane proteomic changes in male and female mouse models of human tauopathy, JNPL3 mice, which could aid in developing treatments for tau-associated neurodegenerative diseases and facilitate the prediction of neuropharmacokinetics in female and male patients.

## Graphical abstract

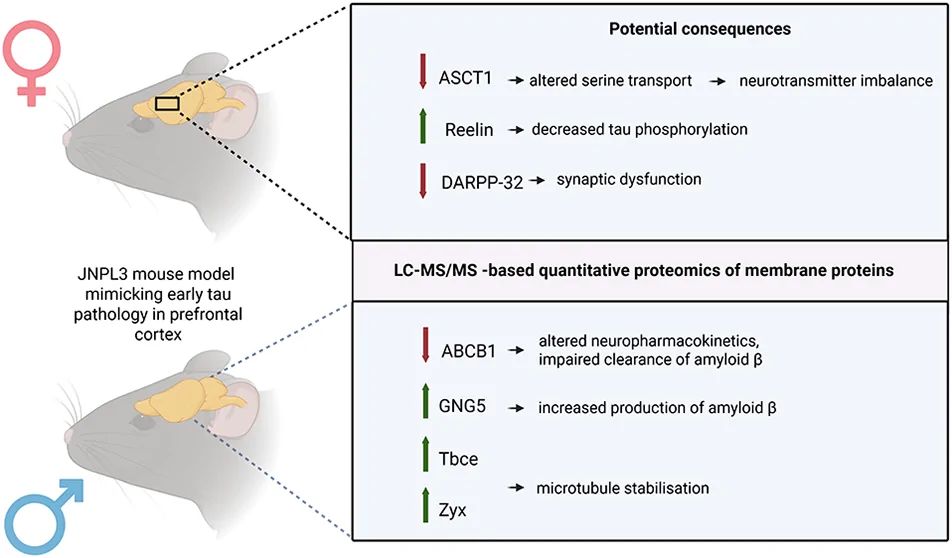

## Introduction

Tauopathies are a heterogeneous group of neurodegenerative disorders caused by tau protein dysfunction and insoluble tau aggregates in the brain.^1,2^ Tauopathies cause a variety of behavioral, memory, language, and movement impairments in patients.^3,4^ In primary tauopathies, such as frontotemporal dementia (FTD) and progressive supranuclear palsy (PSP), tau protein plays a central role in disease pathogenesis. In secondary tauopathies, i.e., Alzheimer’s disease (AD), tau aggregation is assumed to develop in response to other pathogenic events.^5,6^ Tau, encoded by the MAPT (microtubule-associated protein tau) gene, is mainly expressed in neurons, with a predominant location in axons,^7–9^ and at low expression in astrocytes and oligodendrocytes.^10,11^ Under physiological conditions, tau promotes microtubule assembly and stability, contributes to genomic DNA stability, and influences synaptic plasticity and trans-neuronal signaling.^12,13^ In tauopathies, tau hyperphosphorylation leads to tau detachment from microtubules, causing axonal microtubule disassembly, reduction in synaptic vesicles, and postsynaptic dysfunction, contributing to synapse loss.^12,14,15^ Misfolding and oligomerization of insoluble tau deposits into gliofibrillary or neurofibrillary tangles (NFTs) lead to neuronal dysfunction and disease progression. In addition, small tau oligomers are highly neurotoxic and spread from cell to cell in a stereotypical manner across different parts of the brain, causing neuronal and glial toxicity.^16–18^

Various tau-targeting therapies have advanced to over a dozen clinical trials,^19^ while none of the disease-modifying therapies for tauopathies have yet been approved. One of the major therapeutic challenges may be attributed to the limited understanding of biological processes underlying the pathogenesis of tau-associated neurodegenerative diseases. Membrane proteins represent a major part of current drug targets due to their involvement in fundamental cellular processes.^20^ Several membrane proteins have been recognized as key players in tau-associated neurodegenerative disorders.^21,22^ Moreover, membrane proteins, such as receptors and transporters from the solute carrier (SLC) and ATP-binding cassette (ABC) families, play important roles in the movement of solutes, including nutrients and metabolites, as well as in drug delivery to and within the brain.^23,24^ Recently, we reported changes in protein abundances of membrane transporters associated with amyloid pathology in familial models of AD.^25,26^ However, tau-associated alterations in the expression of membrane proteins have not been studied.

The prefrontal cortex, which is responsible for executive functions, including short-term memory, controlling attention, and cognitive flexibility, is affected in many tauopathies, including the early stages of FTD and Pick’s Disease, as well as at the intermediate stage of PSP.^27^ In addition, the levels of pre-filamentous form of tau, i.e., granular tau oligomers, were detected in the prefrontal cortex of pre-symptomatic AD patients already at Braak stage 0 pathology and increased at Braak stage I pathology, indicating that tau triggers the formation of various aggregate types that may contribute to neuronal dysfunction and cell death before NFT formation.^28^ Identifying changes in membrane proteins in the prefrontal cortex at the early stages of tau pathology can provide opportunities to discover novel therapeutic and drug delivery targets to prevent disease progression and associated cognitive dysfunction in tauopathies, including AD.

In the present study, we investigated alterations in membrane protein abundances in the prefrontal cortex of a well-characterized model of tauopathy, male and female JNPL3 mice,^29^ compared to age- and sex-matched wild-type (WT) animals. The JNPL3 mouse model mimics the lesion distribution in some forms of FTD and other human tauopathies, such as PSP, and has been widely used as a tool for studying tau pathology in AD.^29,30^ We explored sensitive liquid chromatography-tandem mass spectrometry (LC-MS/MS)-based quantitative targeted absolute MRM proteomics (QTAP)^25,26^ and data-independent acquisition (DIA) untargeted proteomics. Here, our quantitative proteomics analysis revealed broader proteomic remodeling in male JNPL3 mice, including changes in nucleotide metabolism and translation-related pathways and reduced abundance of the drug efflux transporter ABCB1. These findings suggest that tau pathology may affect membrane protein abundance differently in JNPL3 males and females, potentially influencing disease progression and the drug response.

## Experimental Section

### Animals and Tissue Collection

In the present study, we used female and male 9–9.5-month-old homozygous JNPL3 Tg mice (Tg­(Prnp-MAPT*P301L)­JNPL3Hlmc, stock number 2508) and their appropriate WT control (SW and B6D2F1, stock number 18392), which were purchased from Taconic Biosciences (Rensselaer, NY, USA).^29,31^ The JNPL3 model carries the transgene encoding the human P301L mutation in the *MAPT* gene, with four microtubule-binding repeat domains and no N-terminal inserts (4R/0N).^29^ By 8–10 months, homozygous JNPL3 models were reported to mimic human tauopathies with NFTs in the diencephalon, brainstem, cerebellar nuclei, and spinal cord, consistent with motor neuron loss and development of progressive motor and behavioral disturbances that are comparable in both sexes.^29,32^ The prefrontal cortex function is preserved at the studied age, as no changes in short-term memory were reported at 8 months of age,^32^ while significant impairment was observed by 12–13 months of age in JNPL3 models.^33^ Information about animals is presented in Supporting Table S1.

The animal study was conducted according to the principles and procedures outlined in the Council of Europe Legislation and Regulation for Animal Protection, the ARRIVE guidelines, and the Council of Europe (Directive 86/609) and German laws on animal experiments. All animal experiments were performed at the University of Heidelberg (license number T-15/23, issued by Regierungspräsidium Karlsruhe). Mice were kept on a 12-h light/dark cycle in a humidified and temperature-controlled environment (at a constant temperature of 21 ± 1 °C) with free access to food and water. For tissue isolation, mice were euthanized by gradual-fill carbon dioxide (CO_2_) asphyxiation according to institutional guidelines. CO_2_ euthanasia was selected to avoid the potential confounding effects of anesthetic agents on protein abundance measurements.^34,35^ To minimize any potential impact of CO_2_ exposure on comparisons between experimental groups, all animals were euthanized using the same standardized protocol. Then, transcardiac perfusion with 0.9% NaCl containing Heparin (2500 IU/L, LEO) at 10 mL/min was performed for a total of 5 min per animal. Brains and lumbar spinal cords were collected in separate tubes, immediately snap-frozen, and stored at −80 °C until use.

### Western Blot Analysis

For Western Blot (WB) analysis of the neuron-specific nuclear protein NeuN, we isolated the nuclear fraction from mouse prefrontal cortex (*n* = 7 per group) using the ProteoExtract Subcellular Proteome Extraction Kit (#539790) according to the manufacturer’s protocol. For WB analysis of tau, the prefrontal cortex samples from male and female JNPL3 and WT mice (*n* = 6–7 per group) were weighed and diluted in RIPA lysis buffer (Thermo Fisher Scientific) containing 1× protease and phosphatase inhibitor cocktails (Sigma-Aldrich) at a 1:8 tissue weight/lysis buffer volume ratio. Briefly, the cortical samples were sonicated and centrifuged at 16,000*g* for 1 h at 4 °C. The supernatant was collected and used for WB analysis (hereinafter referred to as “homogenate”). Pierce BCA Protein Assay Kit (Thermo Fisher Scientific) was used to determine the total protein content in the samples. 15 μg from each sample was prepared with loading buffer (1 M Tris-HCl, pH 6.8; 20% sodium dodecyl sulfate SDS; 0.4 μL/mL glycerol; 2 g/L bromophenol blue; and 2 M dithiothreitol) and resolved through SDS-PAGE, except for nuclear fractions, for which 25 μg per sample was loaded. Membranes were blocked in PBS-0.1% Tween 20 + 5% milk for 1 h and incubated overnight with primary antibodies against anti-tau antibody TAU-5 for total tau protein (Total Tau), not phosphorylation-specific (1:5000; #ab80579, Abcam), phospho-tau (Ser202/Thr205) (*p*-tau; 1:1000; #30505S, Cell Signaling Technology), NeuN (1:500; #711054, Thermo Fisher Scientific), Histone H3 (1:1000; #9715, Cell Signaling Technology) and Glyceraldehyde 3-phosphate dehydrogenase (GAPDH; 1:5000; #NB300-327, Novusbio) and with HRP-linked secondary antibody goat anti-rabbit (1:5000; #111-035-144, Jackson ImmunoResearch Labs) and with Anti-rabbit IgG, HRP-linked Antibody (1:2500; #7074P2, Cell Signaling technology), HRP-linked secondary antibody goat anti-mouse (1:5000; #115-035-068, Jackson ImmunoResearch Labs) for 90 min at room temperature. Membranes were visualized using Western Lightning Plus-ECL (PerkinElmer), and images were acquired with a Bio-Rad ChemiDoc imager. Densitometric analysis was performed using Bio-Rad Image Lab software (Version 5.1).

### Quantitative Real-Time Polymerase Chain Reaction (qRT-PCR) Analysis

Gene expression of inflammation markers, i.e., a marker of abnormal activation and proliferation of astrocytes, glial fibrillary acidic protein (*Gfap*), a marker of microglial activation, allograft inflammatory factor 1 (*Aif1*), and an oxidative stress marker, sirtuin-3 (*Sirt3*),^36^ was evaluated by qRT-PCR analysis in the prefrontal cortical tissue of female and male WT and JNPL3 mice (*n* = 6–7 per group) as previously described.^25,37^ RNeasy Mini Kit (Cat#74,004, Qiagen, Stockach, Germany) and Biozym cDNA synthesis Kit (#331475S, Oldendorf, Germany) were used, respectively, for total RNA extraction and cDNA synthesis, according to the manufacturer’s protocols. The PowerUp SYBR Green Master Mix (#A25741, Thermo Fisher Scientific) was used to amplify targets. The gene-specific primers (Thermo Fisher Scientific) are listed in Supporting Table S2. Relative target gene expression was normalized to housekeeping gene β-actin (*Actb*) and calculated as described before.^38^ The qRT-PCR analysis was conducted using the LightCycler 96 (Roche Diagnostics, Mannheim, Germany; 2011) and data acquisition was performed with LightCycler 96 SW 1.1 software, v. 1.1.0.1320 (Roche Diagnostics).

### Absolute Quantification of Transporter Protein Abundances Using LC-MS/MS

In the study, we quantified absolute protein abundances of three ABC transporters, four SLC transporters, LRP1, and the plasma membrane marker Na^+^/K^+^-ATPase in the crude membrane fraction from the prefrontal cortices of female and male JNPL3 and WT mice (*n* = 8–9 per group). The LC-MS/MS-based QTAP analysis samples were prepared according to the previously published protocol.^26^ First, we isolated the crude membrane fraction from the mouse prefrontal cortex using the ProteoExtract Subcellular Proteome Extraction Kit (#539790) according to the manufacturer’s protocol and measured total protein concentrations using the Bio-Rad DC Protein Assay. The crude membrane samples containing 50 μg of total protein were solubilized in 7 M guanidine hydrochloride, 500 mM Tris–HCl (pH 8.5), and 10 mM EDTA. This was followed by protein reduction with dithiothreitol and S-carbamidomethylation with iodoacetamide. The proteins were precipitated with methanol and chloroform, and the resulting precipitates were dissolved in 6 M urea in 0.1 M Tris–HCl (pH 8.5). A five-fold dilution of the samples was done by adding 0.1 M Tris–HCl (pH 8.5) containing internal standard peptides (Supporting Table S3). Lys-C and Protease-Max were added, and the samples were incubated for 3 h at room temperature. Then, the samples were digested at 37 °C for 16 h by adding tosyl phenylalanyl chloromethyl ketone-treated trypsin (enzyme-to-substrate ratio of 1:100). Finally, the samples were acidified with formic acid in water 20% (v/v) and centrifuged at 14,000*g* for 5 min at 4 °C. The resulting supernatants were used for LC-MS/MS-based QTAP analysis and untargeted proteomic analysis.

The LC-MS/MS analysis was performed with an Agilent 6495 triple quadrupole mass spectrometer equipped with an ESI source (Agilent Technologies, Palo Alto, CA, USA) coupled to an Agilent 1290 Infinity LC (Agilent Technologies, Waldbronn, Germany) system. As reported previously, an Advance Bio Peptide Map column (2.1 × 250 mm; 2.7 μm) was used for HPLC separation and elution of the peptides.^26^ Simultaneous peptide detection was performed by applying positive ion MRM mode with a dwell time of 20 ms for each MRM transition. The following parameters were used: source temperature of 210 °C, drying gas flow rate of 16 L/min, nebulizer pressure of 45 psi, and MS capillary voltage of 3 kV. The data were acquired using Agilent MassHunter Workstation Acquisition software, Agilent Technologies, Data Acquisition for Triple Quad., version B.03.01. The data were processed with Skyline software (version 4.1). The target protein quantitation was performed using one unique peptide (Supporting Table S3) as previously described.^26^ Three to four MRM transitions for each peptide linked to the high-intensity fragment ions were used to quantify stable isotope-labeled and unlabeled target peptides. Protein abundance was considered to be under the limit of quantification (ULQ) when only one or two transitions were obtained for the peptide used for the protein quantification. The quantification limits for each target protein were determined as the lowest concentration of a stable isotope-labeled peptide that produced signal peaks with three or four transitions. The protein abundance in crude membrane fractions of the brain prefrontal cortex was presented as absolute values (fmol/μg total protein).

### Untargeted Proteomics Analysis with DIA Approach

The untargeted proteomic analysis of crude membrane fractions from the brain cortex of male and female JNPL3 and WT (*n* = 6–7 per group) mice was performed using a Q-Exactive Focus Orbitrap mass spectrometer (Thermo Fisher Scientific) coupled to a Vanquish UPLC system (Thermo Fisher Scientific) in DIA mode. Chromatographic separation was performed using an Advance Bio Peptide Map column (2.1 × 250 mm; 2.7 μm). A 90 min gradient elution with (A) 0.1% formic acid in water and (B) 0.1% formic acid in acetonitrile was used. The gradient elution was as follows: 0–2 min 2 → 7% of B, 2–5 min 7%, 5–50 min 7 → 30%, 50–83 min 30 → 45%, 83–83.5 min 45 → 80%, 83.5–85.5 80%, 85.5–86 min 80 → 2%, and 86–90 min equilibrium at 2%. A constant flow rate of 0.3 mL/min and a constant column oven temperature of 40°C were used. The injection volume was 20 μl. For the DIA, each cycle was built as follows: one full MS scan at 70,000 resolution with a scan range of 400–900 *m*/*z*, followed by narrow isolation windows of 25 *m*/*z* covering 400–900 *m*/*z* at 35,000 resolution. The AGC target was 3 × 10^6^, and NCE was set to 27.

DIA-NN version 2.1.0 ^39^ was used for spectral library generation, raw spectral data processing, and quantification. The spectral library was generated using SwissProt mouse canonical sequences,^40^ supplemented with two contaminant sets: common mouse tissue contaminants and universal contaminants.^41^ The smart profiling option was applied when the library was generated. This was followed by peptide-spectrum matching, precursor false discovery rate (FDR) estimation, and protein quantification and inference. The precursor FDR threshold was 1%, the number of missed cleavages allowed was set to 1, and precursor charge states were limited to 1–4. Cysteine carbamidomethylation was set as a fixed modification, and oxidation of methionine was allowed as a modification. QuantUMS^42^ high-precision mode was used as the protein quantification strategy.

### Statistical Analysis

The absolute protein abundance and normalized fold mRNA expression are presented as mean ± standard deviation (SD). Statistical significance of differences in absolute protein expression and normalized fold changes in mRNA expression of *Gfap*, *Aif1*, and *Sirt3* in male and female JNPL3 versus age- and sex-matched WT mice was analyzed using an unpaired *t*-test. A *p*-value <0.05 was considered statistically significant. Data analysis was performed in GraphPad Prism, version 9.0.1 (GraphPad Software, San Diego, CA, USA). Protein group data produced by DIA-NN containing proteotypic peptides were analyzed in R version 4.3.3. Minimum-value imputation was performed for each protein to address missing values using the replace_na function with Tidyverse 2.0.0. package.^43^ Statistical analyses were performed with LIMMA (Linear Models for Microarray and RNA-Seq Data) version 3.58.1.^44^ Linear models were implemented using the lmFit function for each log 2-transformed protein abundance, followed by empirical Bayes moderation to reduce variance using the eBayes function. Moderated *t*-tests were performed with the topTable function for differential protein abundance analysis between the groups, and the *p*-values were FDR-adjusted using the Benjamini-Hochberg method.^45,46^ Log 2-fold changes were obtained for each protein in addition to the adjusted *p*-values, which were considered statistically significant when less than 0.05.

## Results

### Characterization of Female and Male JNPL3 Mouse Models of Human Tauopathy

The JNPL3 mouse model has been extensively characterized previously.^29^ In this study, we confirmed the early stage of tau pathology in the prefrontal cortex in female and male JNPL3 mice at 9–9.5 months of age by Western blotting. Both female and male JNPL3 mice demonstrated a 5.5-fold increase in average total tau abundance in the prefrontal cortical homogenate (Figures 1A–D and S1A,B) as compared to sex-matched WT mice, indicating similar abundance of human tau protein in both sexes in the JNPL3 model as compared to WT mice. In addition, a comparable significant increase in p-tau abundance in the prefrontal cortical homogenate was observed in female (2.8-fold) rather than in male (2.3-fold) JNPL3 mice as compared to sex-matched WT, respectively (Figures 1A–D and S1A,B).

**Figure 1:**
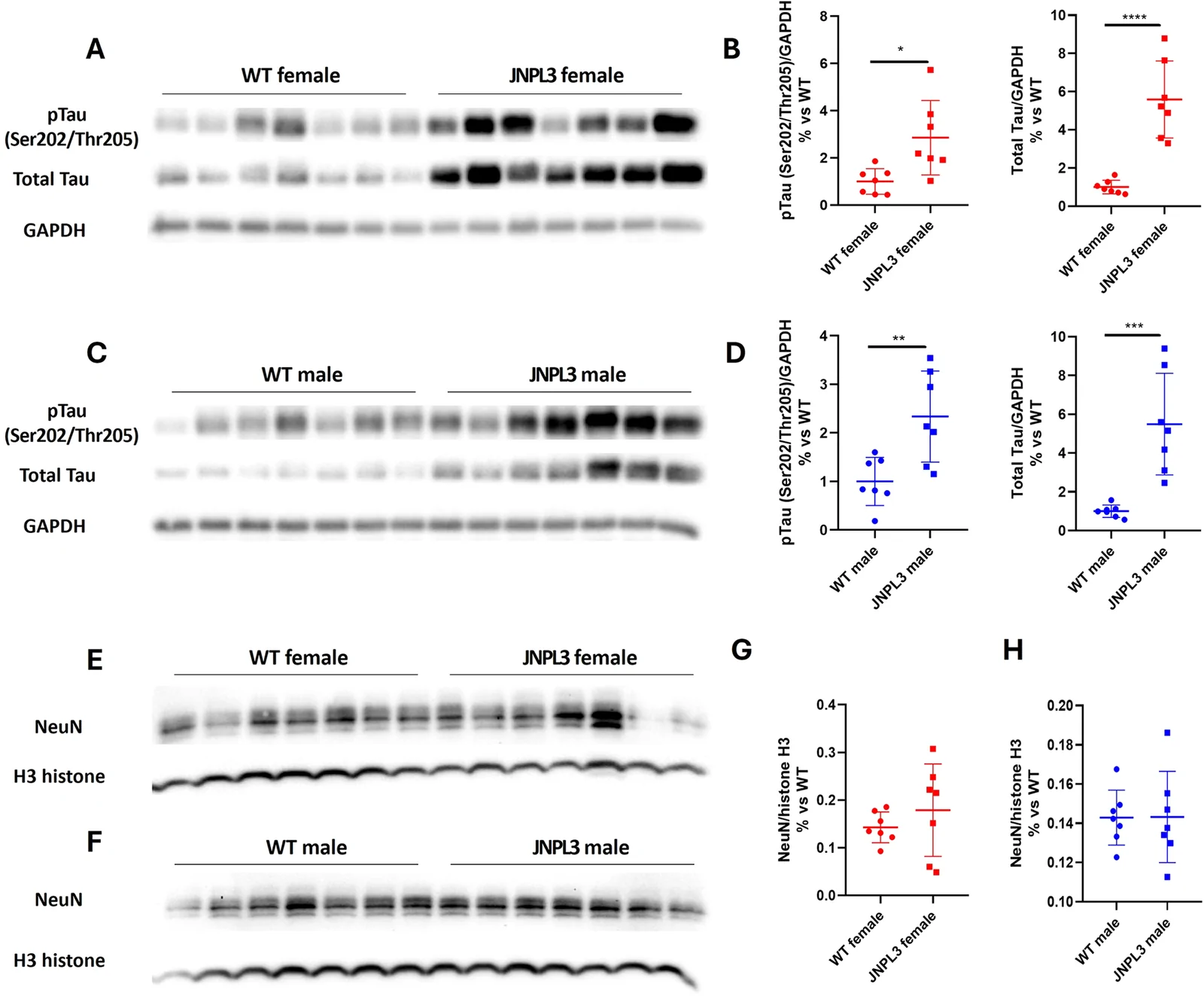
(A–D) Representative WB and relative densitometries of *p*-tau and total Tau protein abundance in brain cortical homogenate samples from female (A, B) (*n* = 7) and male (C, D) (*n* = 7) JNPL3 mice. *p*-tau and total Tau abundance were quantified as the ratio to GAPDH, used as a loading control. Representative WB and relative densitometries of NeuN protein abundance in nuclear fractions from brain cortical samples from female (E, G) (*n* = 7) and male (F, H) (*n* = 7) JNPL3 mice. NeuN abundances were quantified as the ratio of NeuN to histone H3, used as a loading control. All the data are presented as mean ± SD. The statistical significance of differences between groups was analyzed using an unpaired *t*-test. Statistically significant differences are marked with asterisks, where * *p* < 0.05; ** *p* < 0.01; *** *p* < 0.001; **** *p* < 0.0001.

To evaluate the effect of tau pathology on synaptic integrity and neuronal loss, we investigated the changes in the abundance of NeuN, a neuronal marker, in the nuclear fractions of the prefrontal brain cortex in female (Figures 1E,G and S1C) and male (Figures 1F,H and S1D) JNPL3 mice versus sex-matched WT animals, with no significant differences observed between the groups. To assess potential changes in synaptic membrane proteins, the abundance of Dlg4 (known as Postsynaptic density protein 95), Vesicular glutamate transporters 2 and 1 (encoded by *Slc17a6*, *Slc17a7*), and Synaptophysin (Syp) were compared in both female and male mice JNPL3 mice versus sex-matched WT animals using our DIA dataset (Supporting Figure S2). No significant differences in protein abundance of Dlg4, Slc17a6, Slc17a7, and Syp were observed between the models (Supporting Figure S2). Finally, the study of gene expression of inflammation markers, i.e., *Gfap* and *Aif1*, as well as the oxidative stress marker *Sirt3*, has not revealed any changes in their normalized mRNA expression in female and male JNPL3 mice compared to sex-matched WT animals (Supporting Figure S3).

### Absolute Protein Abundances of Transporters in the Prefrontal Brain Cortices of JNPL3 Mice

To assess the impact of tau pathology on protein abundances of transporters in the brain parenchymal cells, we quantified three ABC transporters, four SLC transporters, and low-density lipoprotein receptor-related protein 1 (LRP1) in the brain cortical tissues of female and male WT and JNPL3 mice (Figure 2 and Supporting Table S1).

**Figure 2:**
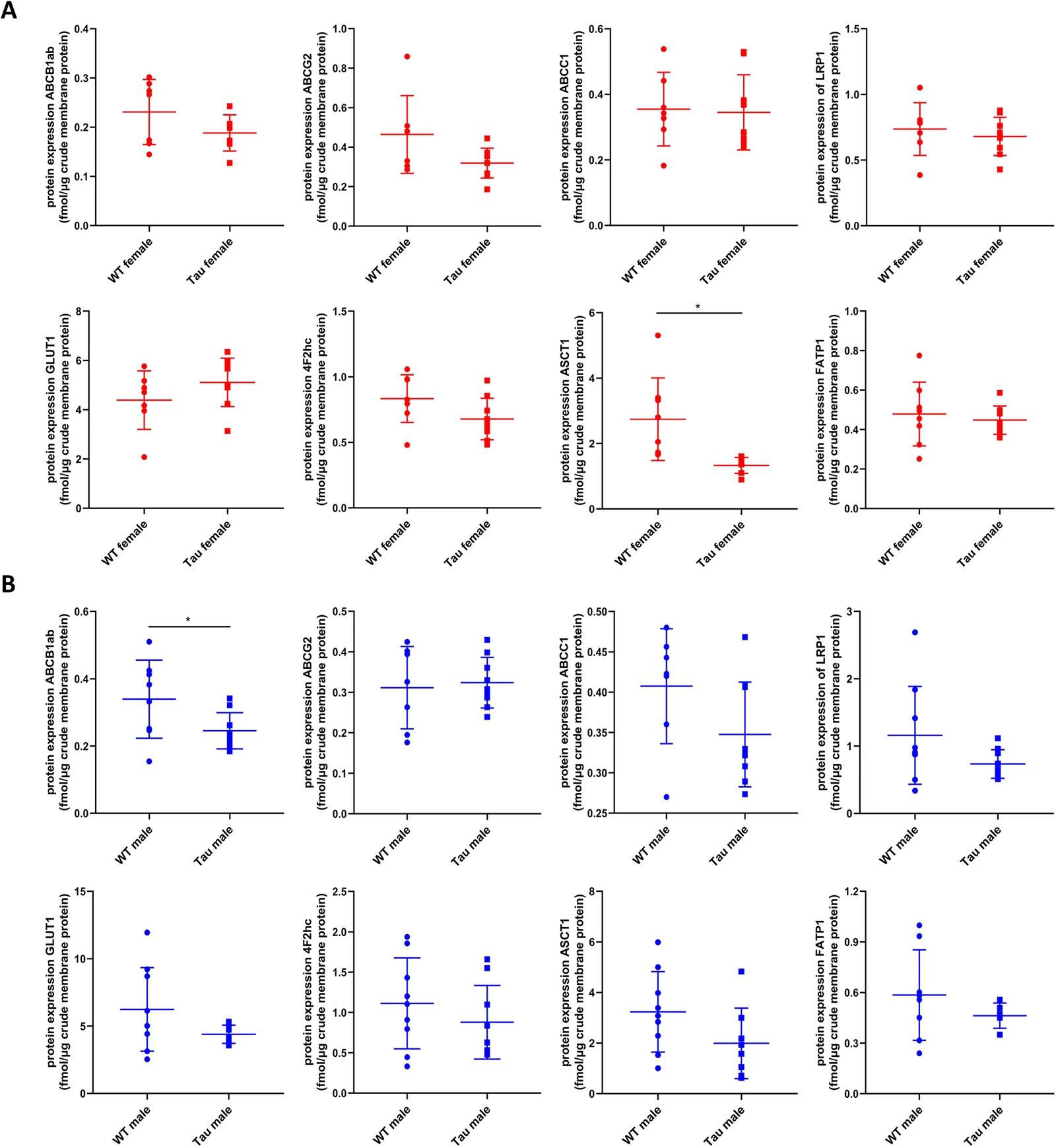
Comparison of absolute protein abundances of ABC and SLC transporters (fmol/μg total protein) in the crude membrane fraction of the brain prefrontal cortex from female (A) and male (B) JNPL3 mice (*n* = 8–9) versus age- and sex-matched WT mice (*n* = 8–9). The line represents the group means, and whiskers represent the SD. The statistical significance of changes in protein abundances between groups was analyzed by an unpaired *t*-test. Statistically significant differences are marked with asterisks, where * indicates *p* < 0.05.

We found significantly lower absolute ABCB1 protein abundance in male JNPL3 mice (*p* = 0.04) compared to sex-matched WT mice, with no differences in female groups. The absolute protein abundance of alanine/serine/cysteine/threonine transporter 1 (ASCT1) was significantly lower in female JNPL3 mice (*p* = 0.01) and did not differ between male groups (Figure 2). No changes were observed in the protein abundance of LRP1, glucose transporter 1 (GLUT1), 4F2 cell-surface antigen heavy chain (4F2hc), and long-chain fatty acid transport protein 1 (FATP1) (Figure 2). Importantly, there were no significant differences in the protein expression of the membrane marker Na+/K+-ATPase between the study groups, indicating similar enrichment of the extracted membrane fraction between the samples (Supporting Table S1).

### Global Changes in Membrane Protein Abundance in the Prefrontal Brain Cortices of JNPL3 Mice

The DIA-based untargeted protein quantification was performed using DIA-NN (v2.1.0) on raw LC-MS/MS data from membrane fractions of prefrontal cortices of male and female JNPL3 and WT mice. Across the brain cortex samples from all animals, DIA-NN identified 4649 unique protein groups and 54,686 peptides using the spectral library generated with an integrated tool. Principal component analysis (PCA) performed in R on minimum-value imputed and log 2-transformed data revealed a tighter clustering of JNPL3 samples according to sex; however, this separation is less evident in the WT group (Figure 3A). The separation observed in the PCA was statistically supported by differential protein abundance analysis using the Limma package on the same dataset, which identified significant proteomic changes between sexes in the JNPL3 groups (Figure 3C,D,F) with no differences in male and female WT mice (Figure 3E). These findings indicate that tau pathology in JNPL3 mice leads to sex-specific proteomic alterations that are absent under baseline conditions in WT mice, highlighting a disease-dependent interaction between the sex and molecular pathways.

**Figure 3:**
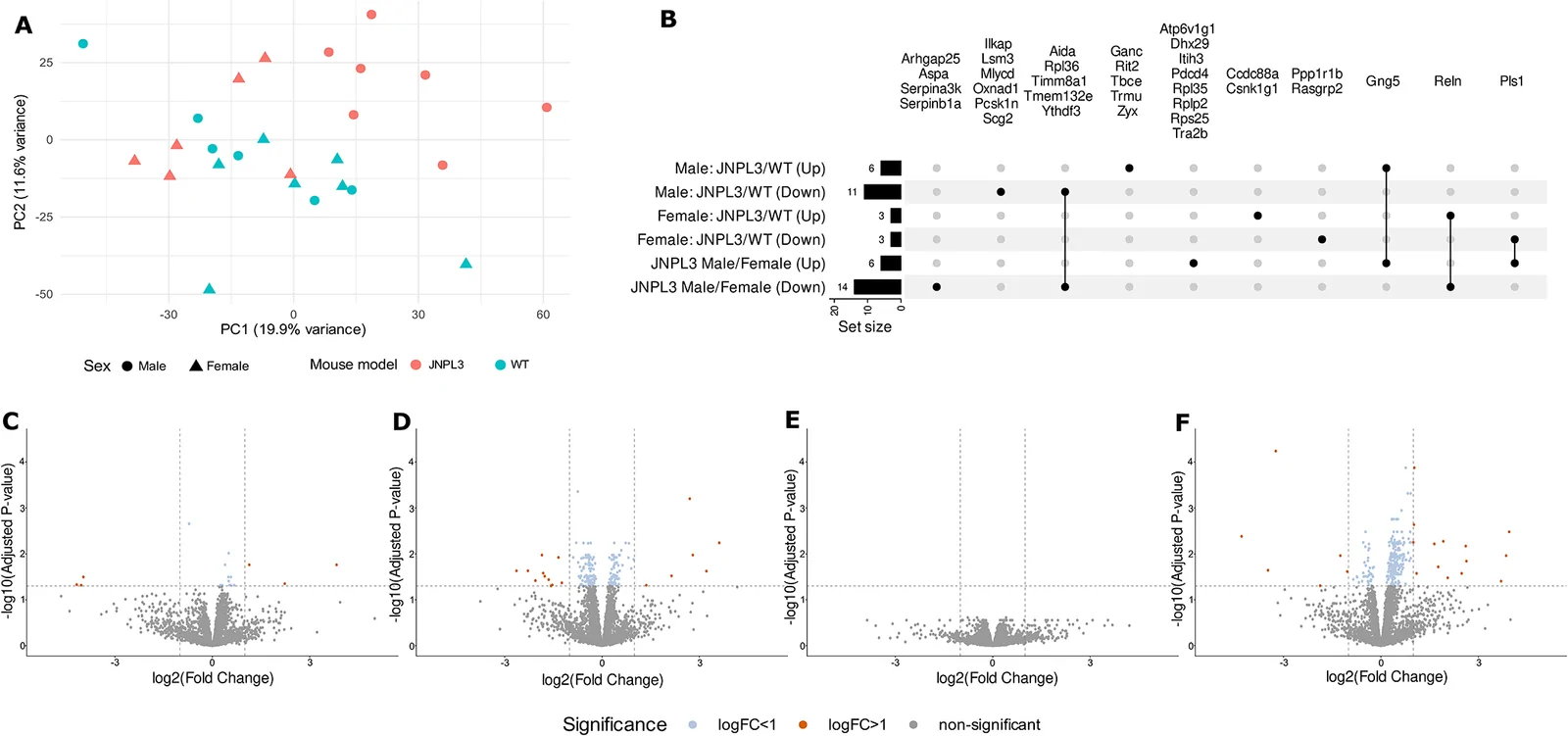
Differences between JNPL3 and WT mice of both sexes in mouse protein abundances based on untargeted proteomics analyses (*n* = 6–7 per group). (A) The principal component analysis of log 2-transformed mouse protein abundances for visualization of variance across the groups. Dots and triangles represent individual male and female mice, respectively. Red color indicates the JNPL3 group and turquoise the WT group. (B) UpSet plot illustrating the overlap of significantly altered protein abundances (log 2-fold change > ±1, adjusted *p*-value < 0.05) between male and female JNPL3 and WT mice. The horizontal bars on the left indicate the total number of differentially abundant proteins in each comparison (set size). The matrix of solid black circles and connecting lines denotes the specific intersections between the groups. The identities of the overlapping proteins are listed directly above the corresponding columns. (C, D) Volcano plots showing differentially abundant proteins in female (C) and male (D) JNPL3 mice compared to sex-matched wild-type controls; in WT male mice compared to WT female mice (E); in male JNPL3 mice compared to JNPL3 female mice (F). Significance thresholds correspond to adjusted *p*-value < 0.05 (horizontal dotted line) and log 2-fold change of > ±1 (vertical dotted lines). Orange dots represent significant alterations with log 2-fold change > ±1. Light blue dots indicate significant alterations with log 2-fold change < ±1.

Comparative analysis revealed increased abundance of 14 proteins and reduced abundance of 4 proteins in JNPL3 female mice compared to the WT female mice (adjusted *p* < 0.05) (Figure 3B,C and Table S5). After applying a log 2-fold change (log 2FC) threshold of >±1 (corresponding to doubling or halving protein quantities), six of these proteins remained, showing higher (Ccdc88a, Reln, Csnk1g1) or lower (Pls1, Ppp1r1b, Rasgrp2) protein abundances in JNPL3 female mice compared to the WT female mice (Figures 3B,C and 4). In male JNPL3 mice, the abundances of 74 proteins were significantly higher, and the abundances of 100 proteins were significantly lower (adjusted *p* < 0.05) as compared to the WT male mice (Figure 3B,D and Table S4). After applying the log 2FC > ±1 threshold, six proteins were found to have higher abundance (Rit2, Zyx, Gng5, Ganc, Tbce, Trmu) and 11 proteins (Rpl36, Lsm3, Scg2, Tmem132e, Ythdf3, Aida, Ilkap, Oxnad1, Mlycd, Pcsk1n, Timm8a1) had lower abundance in the membrane fraction of the brain cortex of male JNPL3 mice versus male WT mice (Figures 3B,D and 4). While comparing JNPL3 mouse models, 14 proteins (Rpl35, Rps25, Rplp2, Dhx29, Timm8a1, Tmem132e, Rpl36, Atp6v1g1, Itih3, Aida, Ythdf3, Pdcd4, Tra2b, Reln) had lower abundance and 6 proteins (Pls1, Serpina3k, Gng5, Arhgap25, Aspa, Serpinb1a) had higher abundance in male JNPL3 mice compared to female JNPL3 mice (adjusted *p* < 0.05, log 2FC > ±1) (Figure 3B,F and Table S6).

**Figure 4:**
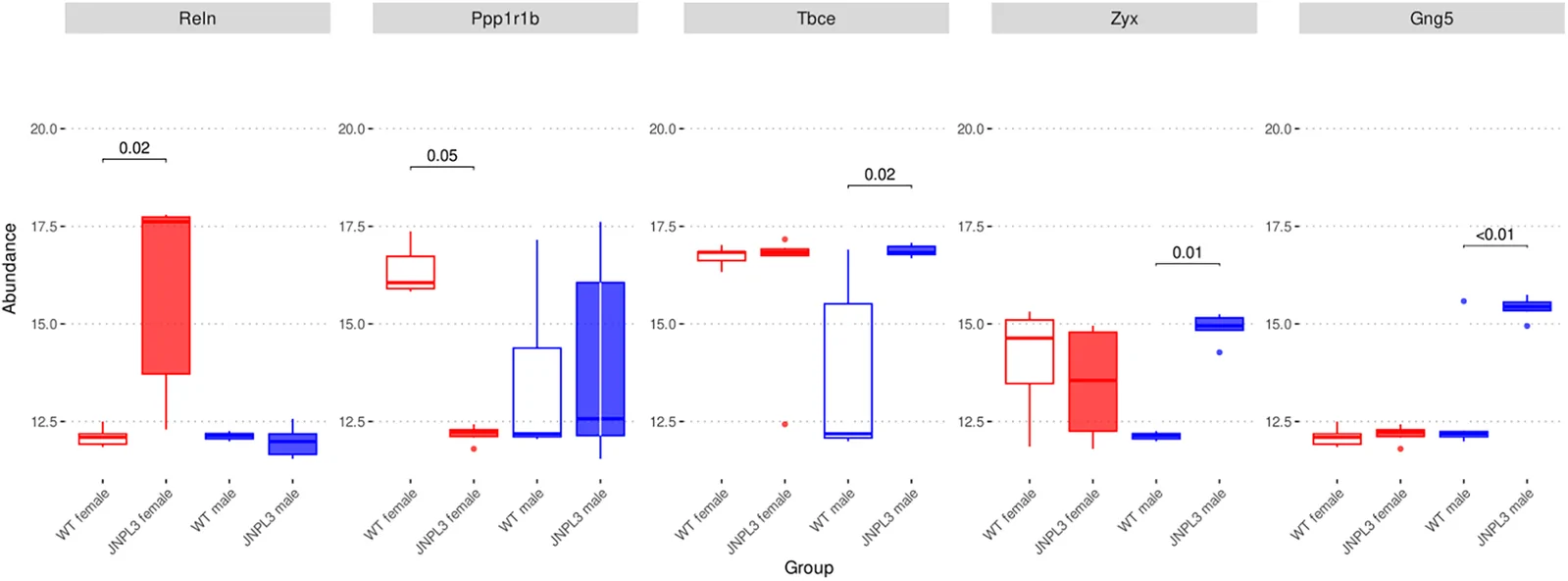
Normalized and transformed abundances of selected proteins, including mouse Reln, Ppp1r1b, Zyx, Tbce, and Gng5, presented as boxplots, grouped by sex and genotype. Data for female mice are colored red, and those for male mice are colored blue. Filled boxes represent the JNPL3 mice, while empty boxes represent the WT mice. Brackets above the plots indicate statistically significant differences, defined as an adjusted *p*-value < 0.05 and an absolute log 2-fold change (log FC) greater than 1.

Pathway enrichment analysis using the Reactome database (release 92) was performed on the set of significantly altered mouse proteins (adjusted *p*-value < 0.05) without a log 2FC cutoff, because biological pathways are often characterized by coordinated changes across multiple proteins, including proteins exhibiting modest effect sizes. Retaining all statistically significant proteins increased the input list size and improved the ability to detect biologically relevant pathway-level alterations. In male JNPL3 mice, significant changes (FDR < 0.05) were identified in several pathways as compared to the sex-matched WT mice, including upregulated metabolism of nucleotides and interconversion of nucleotide di- and triphosphates (Figure 5A and Table S8) as well as multiple downregulated pathways involved in translation and ribosomal formation and function (Figure 5B and Table S9). The analysis identified upregulated mitochondrial translation pathways in female JNPL3 mice compared with female WT mice (Figure 5C and Table S10). Given the small number of significantly downregulated proteins in females, no enrichment of the downregulated pathways is reported.

**Figure 5:**
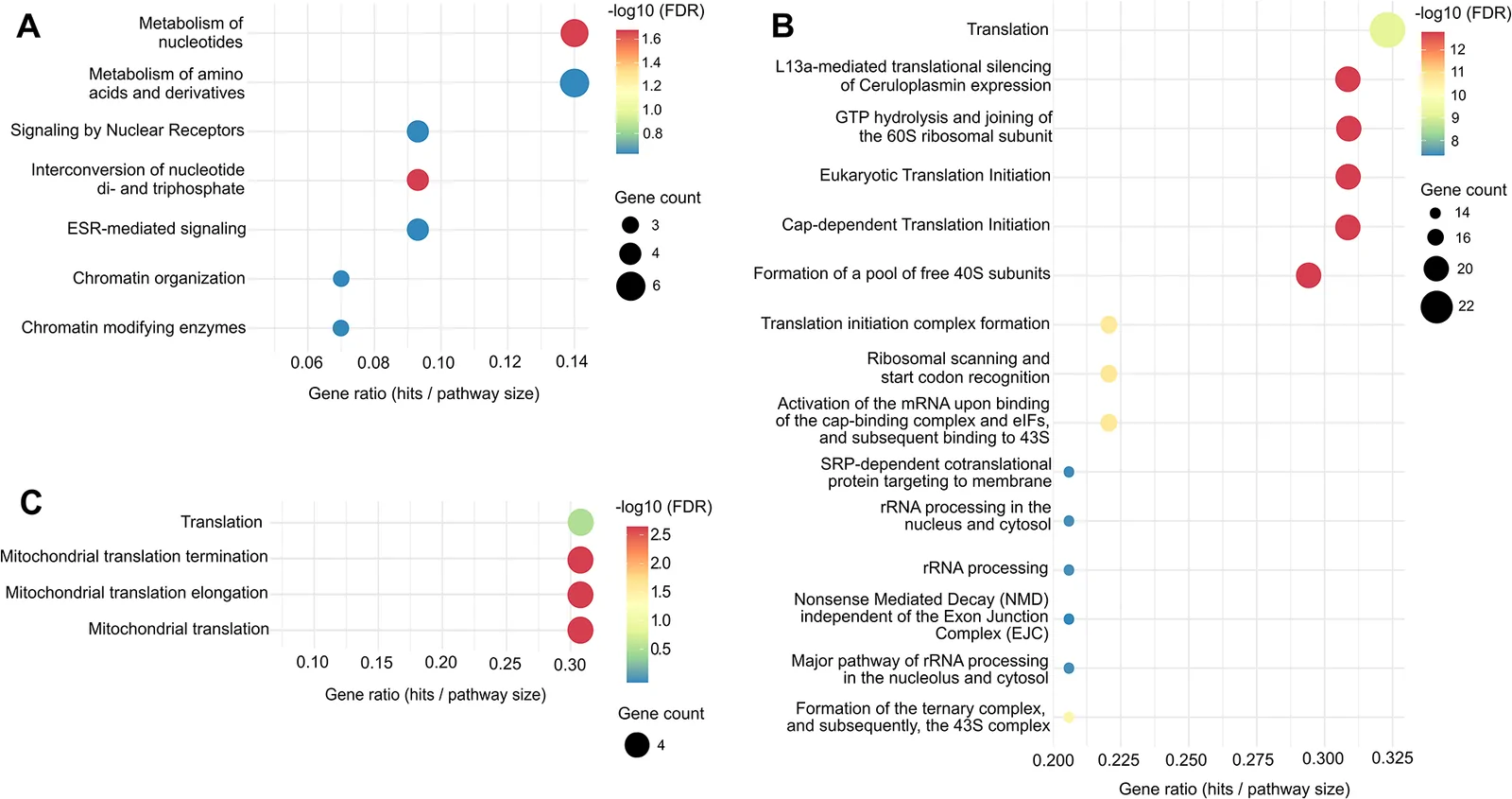
(A, B) Upregulated (A) and downregulated (B) pathways in male JNPL3 mice compared to the sex-matched WT animals. (C) Upregulated pathways in JNPL3 female mice compared to sex-matched WT mice. The larger dot size indicates a greater number of altered proteins in the pathway. The enrichments with FDR < 0.05 are considered significant (>1.301 in the −log 10 scale). The dot color indicates significance, with red denoting the greatest significance and blue the lowest. The Reactome database pathway enrichment analysis was performed using the significantly altered (adjusted *p*-value < 0.05, all fold changes) proteins in the JNPL3 mice compared to the sex-matched WT mice.

## Discussion

The development of disease-modifying treatments for tauopathies remains challenging due to the complex pathogenesis of tau-associated diseases. In addition, the presence of sex differences in tauopathies can hinder the development of therapeutic approaches. Previous studies demonstrated that tau-targeting therapies are more effective in the early stages of tau pathology.^32,47^ Here, we studied changes in membrane protein abundances in the prefrontal cortex, which is affected in several tauopathies and associated with cognitive impairments, in 9–9.5-month-old male and female mouse models of tauopathy, JNPL3, mimicking early stages of tau pathology in the prefrontal cortex. Thus, we aimed to discover altered proteins that can serve as potential preventive therapeutic targets for tau-associated diseases, as well as alterations in the protein abundance of transporters and receptors involved in drug delivery, with consideration of sex differences in this model.

We confirmed the early development of tau pathology in the prefrontal cortex in the studied model. The comparable increases in abundances of total tau and phosphorylated tau in male and female JNPL3 mice compared with sex-matched WT animals indicated an equal pool of tau in both sexes in JNPL3 mice. Importantly, the observed tau pathology in the prefrontal cortex of male and female JNPL3 mice did not result in synaptic or neuronal loss, inflammation, or oxidative stress, as indicated by the absence of changes in the corresponding markers compared with sex-matched WT animals. Overall, based on previous reports^29,32^ and these data, we assume that male and female JNPL3 mice at the study age exhibit early pretangle pathology in the prefrontal cortex without functional changes in this brain region. Therefore, changes in membrane proteins in the male and female JNPL3 mouse models described below likely reflect sex-associated responses to tau pathology.

The expression and function of transporters and receptors have been extensively studied in the context of AD, but not other tauopathies.^48–50^ In the present study, we quantified for the first time the absolute membrane protein abundance of several ABC and SLC transporters and LRP1 in cortical tissues from female and male JNPL3 mice and sex-matched WT animals. First, we investigated sex-specific changes in the abundance of ABC transporters (ABCB1, ABCG2, and ABCC1) and LRP1, which are thought to be involved in the clearance of amyloid β (Aβ) from the AD brain, thereby playing a critical role in the progression of AD.^50–52^ Among those transporters, we observed a sex-associated significant decrease in ABCB1 protein abundance in male JNPL3 mice compared to sex-matched WT animals, with no differences in female groups. ABCB1 transporter, also known as P-glycoprotein (P-gp) or multidrug resistance protein 1 (MDR1), is an ATP-dependent efflux pump expressed at the brain capillary endothelial cells, pericytes, astrocytes, and neurons.^49^ It plays a crucial role in maintaining CNS homeostasis by transporting toxins, drugs, and metabolic waste products out of the brain parenchyma.^53,54^ To date, the impact of tau pathology on ABCB1 abundance has not been studied. However, reduced ABCB1 functional expression may alter neuropharmacokinetics and impair the clearance of neurotoxic compounds, including Aβ, contributing to a toxic brain microenvironment that promotes tau hyperphosphorylation and aggregation.^55,56^ Individuals with AD exhibit reduced ABCB1 activity at the blood-brain barrier (BBB) relative to healthy older individuals.^57^ Additionally, ABCB1 abundance was significantly lower in prefrontal cortex and hippocampal brain samples from AD patients compared to non-demented individuals.^58,59^ The changes in functional expression of ABCB1 in patients with tauopathies and the potential impact of these alterations on neuropharmacokinetics should be further investigated.

A significant decrease in the ASCT1 protein abundance was observed only in female JNPL3 mice, but not male JNPL3 mice, as compared to age- and sex-matched WT animals. ASCT1 (encoded by *Slc1a4*) is a neutral amino acid transporter that mediates the import of l-serine into brain cells and the efflux of d-serine, a co-agonist of NMDA receptors.^25,60^ Previously, we reported significantly higher ASCT1 abundance in female 5xFAD mice compared with WT animals, with no change in male 5xFAD mice, and these were associated with higher brain cortical serine levels in female 5xFAD mice.^25^ This study revealed an opposite trend compared with 5xFAD mice, a mouse model mimicking Aβ pathology, thus indicating various impacts of tau and Aβ pathology on ASCT1 protein abundance.

Proteome-wide identification of drug targets in tau-associated neurodegenerative diseases in men and women remains understudied. Our untargeted DIA proteomics analysis revealed broader membrane proteomic remodeling in male JNPL3 mice than in female JNPL3 mice compared to sex-matched WT animals. Distinct alterations in membrane proteins associated with various pathways were observed in female and male JNPL3 mice compared with sex-matched WT mice. Thus, marked upregulation of pathways related to nucleotide metabolism and interconversion of nucleotide di- and triphosphates was observed in male JNPL3 mice compared to WT mice, concurrently with downregulation of pathways associated with translation and ribosomal formation/function. Previous studies have demonstrated a link between tau pathology and reduced protein synthesis, accompanied by reduced ribosomal protein abundances.^61,62^ Thus, pathological tau impairs the cell’s ability to translate proteins and simultaneously upregulates nucleotide metabolic flux, likely as a compensatory mechanism to manage cellular stress. Taken together, this discrepancy may reflect a shift from a “growth and maintenance” state to a “repair and stress response” phase in males, in which nucleotide and nucleic acid homeostasis takes priority over protein synthesis under pressure. In female JNPL3 mice, our study revealed upregulation in mitochondrial translation pathways. There is extensive documentation of mitochondrial dysfunction in tauopathies.^63^ This upregulation might be an early adaptive response to mitigate mitochondrial impairment due to increased mitochondrial stress and increased energy demand. Overall, delayed but broader stress response in male JNPL3 mice likely reflects a later-stage activation of protective mechanisms, contrasting with earlier mitochondrial adaptations in JNPL3 females.

Our study revealed several proteins associated with tau pathology that were altered in a sex-specific manner in JNPL3 mice and have previously been reported as drug targets for various diseases. Thus, reelin (Reln), an extracellular glycoprotein involved in synaptic plasticity through interactions with receptors on the neuronal plasma membrane, is of particular interest in the context of tauopathies. Studies have shown increased *Reln* transcriptional activity and higher protein abundance of Reln in the CSF and frontal cortex of AD patients, as well as in CSF from FTD and PSP patients.^64,65^ Since overexpression of Reln in tauopathy mice (Tg4; pThy1-Tau-VLW mice) has been shown to reduce tau phosphorylation and improve synaptic plasticity,^66^ we can assume that the increased protein abundance observed in female JNPL3 mice might be a compensatory mechanism to reduce tau hyperphosphorylation and synaptic/cognitive deficits. Modulating Reelin signaling to suppress kinase-driven tau hyperphosphorylation was reported as a potential therapeutic approach.^66^ Thus, future studies should investigate the potential of targeting Reelin signaling at the early stages of tauopathy with a consideration of sex differences.

In addition, we observed reduced abundance of dopamine- and cAMP-regulated neuronal phosphoprotein, 32 kDa (DARPP-32, encoded by the *PPP1R1B* gene), which is an inhibitor of protein phosphatase-1 (PP-1), a key mediator of tau dephosphorylation.^67^ In addition, DARPP-32 is involved in dopaminergic signaling and in synaptic functions,^68,69^ and its reduced levels have been observed in AD patients, leading to altered synaptic plasticity, disrupted signal integration, and contributing to behavioral deficits.^68^ The authors proposed that synaptic dysfunction and cognitive impairment result from destabilization of the PP1–DARPP-32 complex and dysregulation of CREB signaling due to DARPP-32 degradation, leading to increased free PP-1 levels. Due to its important role in dopamine and glutamate signaling, DARPP-32 has been studied as a promising drug target for treating a spectrum of dopamine-related conditions.^70–72^ Therefore, our data in female JNPL3 mice support targeting DARPP-32–PP1 at early stages of tau pathology to prevent synaptic dysfunction.

In male JNPL3 mice, we observed a sex-specific increase in protein abundance of tubulin-specific chaperone E (encoded by *Tbce*). Alterations in Tbce protein abundance disrupt tubulin homeostasis, leading to the accumulation of phosphorylated tau and the progression of tau pathology.^73,74^ Another protein with increased protein abundance in male JNPL3 mice is zyxin (Zyx), a membrane-associated protein that regulates cytoskeletal remodeling and stability, neuronal information transmission, morphology, and synaptic function.^75–77^ The increased protein abundances of Tbce and Zyx observed in male JNPL3 mice may suggest a compensatory strategy to stabilize microtubules and a protective action to maintain cytoskeletal integrity and facilitate rearrangement in the initial or prodromal stages of tauopathy, when functional deficits are still reversible.^76^ Interestingly, we observed higher protein abundance of the homotrimer G protein subunit γ-5 (Gng5) in male JNPL3 mice compared with WT animals, but not in females. Importantly, GNG5 was shown to be upregulated in the brain of AD patients, where it regulates Aβ_42_ production by directly interacting with the γ-secretase catalytic subunit presenilin 1.^78^ Consequently, in the advanced stages of the disease, when signaling pathways are profoundly altered and Aβ production contributes to neurodegeneration, Gng5 represents a highly attractive target. Intervening on Gng5 at this stage could help reduce amyloid burden and mitigate the synergistic effects between tau and amyloid pathology.

It is important to acknowledge the limitations of this study. As mentioned above, the analysis was confined to a single time point, limiting the evaluation of the temporal evolution of protein abundances and any sex-related differences that may arise during tau pathology progression in female and male models. Another limitation of the study is that phosphorylation-specific changes were not investigated. Future phosphoproteomic analyses may provide additional insights into signaling pathways and phosphorylation-dependent mechanisms associated with tau pathology in the JNPL3 mice. In addition, to enrich the membrane proteins, we investigated changes in protein abundances in the crude membrane fraction, which includes the plasma membrane and membranes of other organelles. Therefore, the results of the study provide cumulative information about proteins localized in the plasma membrane and in the membranes of mitochondria, the endoplasmic reticulum, and so on. In addition, sex-related differences in transporter and receptor protein abundances were observed in the brain cortical tissue, representing parenchymal cells. Future studies are planned to explore the changes in the BBB proteins in more detail.

In conclusion, the present study revealed distinct membrane proteomic changes in the prefrontal cortex of female and male JNPL3 mice, mimicking the early stages of tau pathology in this brain region. Our quantitative MRM proteomics analysis revealed a significant decrease in the protein abundance of the drug efflux transporter ABCB1 in male JNPL3 mice but not in female JNPL3 mice, as compared to sex-matched WT animals. Moreover, the DIA proteomics analysis identified multiple differentially abundant proteins involved in various pathways in male and female JNPL3 mice, which could serve as candidates for the development of novel therapeutic approaches for tauopathies in the future. Further studies to investigate the time course of membrane proteomic changes as well as their functional expression in patients with tauopathies would improve our understanding of their roles in tau pathology and confirm their potential use for therapeutic targeting.

## Supplementary Material





## Data Availability

DIA proteomic data are available from the ProteomeXchange Consortium database, permanent link https://panoramaweb.org/P0Grv5.url; ProteomeXchange ID is PXD070169.

## Notes

Characteristics of WT and JNPL3 mice, as well as individual absolute protein abundances of investigated transporters (fmol/μg total protein) in the crude membrane fraction of the brain cortical tissue of female and male WT and JNPL3 mice (*n* = 8–9) (Table S1); primer sequences for qRT-PCR analysis (Table S2); probe peptide amino acid sequences and multiple-reaction monitoring transitions for the LC-MS/MS-based quantitative targeted absolute proteomics analysis (Table S3); original Western blots of p-tau, Total tau, and NeuN protein abundances in brain cortical samples from the whole homogenates of female JNPL3 mice (*n* = 7) and male JNPL3 mice (*n* = 7) compared to age-matched WT (Figure S1); normalized and transformed abundances of neuronal membrane proteins, including mouse Dlg4, Slc17a6, Slc17a7, and Syp, presented as boxplots, grouped by sex and genotype (Figure S2); and comparison of gene expression of glial fibrillary acidic protein (Gfap), allograft inflammatory factor 1 (Aif1), and an oxidative stress marker sirtuin-3 (Sirt3) in the brain cortical tissue of female and male JNPL3 mice vs. sex-matched WT mice (*n* = 6–7 per group) (Figure S3) (PDF)
Comparison of the protein abundances in the crude membrane fraction of the brain cortex in male JNPL3 mice (*n* = 7) and WT mice (*n* = 6) (Table S4); comparison of the protein abundances in the crude membrane fraction of the brain cortex in female JNPL3 mice (*n* = 6) and WT mice (*n* = 7) (Table S5); comparison of the protein abundances in the crude membrane fraction of the brain cortex in female JNPL3 mice (*n* = 6) and male JNPL3 mice (*n* = 7) (Table S6); comparison of the protein abundances in the crude membrane fraction of the brain cortex in male wild-type mice (*n* = 6) compared to the female wild-type mice (*n* = 7) (Table S7); reactome pathway enrichment based on increased protein abundances in male JNPL3 mice compared to WT mice (Table S8); reactome pathway enrichment based on decreased protein abundances in male JNPL3 mice compared to WT mice (Table S9); and reactome pathway enrichment based on increased protein abundances in female JNPL3 mice compared to WT mice (Table S10) (XLSX)
