# Supplementary material for: Altered Membrane Protein Abundance in the Cerebral Cortex of the JNPL3 Mouse Model of Tauopathy
Source: J Proteome Res. 2026 Sep 16;25(10):5599–610. doi: 10.1021/acs.jproteome.6c00679 (PMC13640504; doi:10.1021/acs.jproteome.6c00679)
Supplement: Supplementary file 1 [file pr6c00679_si_001.pdf]

Altered membrane protein abundances in the cerebral cortex of the JNPL3 mouse model of tauopathy

Sabrina Petralla[1], Jooseppi Hyvärinen[2], Mikko Gynther[3], Alexey Afonin[2], Marko Lehtonen[3], Seppo Auriola[3], Maria Panayotova[1,4], Katja M. Kanninen[2], Gert Fricker[1], Elena Puris[1,2]*

[1]Institute of Pharmacy and Molecular Biotechnology, Ruprecht-Karls-University, Heidelberg, DE-69120, Germany
[2]A.I. Virtanen Institute for Molecular Sciences, University of Eastern Finland, Kuopio, FI-70211, Finland
[3]School of Pharmacy, University of Eastern Finland, Kuopio, FI-70211, Finland
[4]Institute of Pharmacology, Faculty of Medicine, Heidelberg University, Heidelberg, DE-69120, Germany

Table of contents

Supplementary Tables

Table S1. Characteristics of WT and JNPL3 mice, as well as individual abundances of investigated proteins (fmol/µg total protein) in the crude membrane fraction of the brain cortical tissue of WT and JNPL3 mice……………………………………………………………………S2
Table S2. Primer sequences for qRT-PCR analysis………………………………………………S3
Table S3. Probe peptide amino acid sequences and multiple reaction monitoring transitions for the LC-MS/MS-based quantitative targeted absolute proteomics analysis…………………………...S4
Table S4. Comparison of the protein abundances in the crude membrane fraction of the brain cortex in male JNPL3 mice and WT mice (XLSX).
Table S5. Comparison of the protein abundances in the crude membrane fraction of the brain cortex in female JNPL3 mice and WT mice (XLSX).
Table S6. Comparison of the protein abundances in the crude membrane fraction of the brain cortex in female JNPL3 mice and male JNPL3 mice (XLSX).
Table S7. Comparison of the protein abundances in the crude membrane fraction of the brain cortex in male wild-type mice compared to female wild-type mice (XLSX).
Table S8. The Reactome pathway enrichment based on increased protein abundances in male JNPL3 mice compared to WT mice (XLSX).
Table S9. The Reactome pathway enrichment based on decreased protein abundances in male JNPL3 mice compared to WT mice (XLSX).
Table S10. The Reactome pathway enrichment based on increased protein abundances in female JNPL3 mice compared to WT mice (XLSX).

Supplementary Figures

Figure S1. Original Western blots of p-tau, Total tau, and NeuN protein abundances in brain cortical samples from whole homogenates of brain cortical tissue of female and male JNPL3 mice vs. sex-matched WT mice……………………………………………………………………S5
Figure S2. Normalised and transformed abundances of neuronal membrane proteins, including mouse Dlg4, Slc17a6, Slc17a7, Syp, in the brain cortical tissue of female and male JNPL3 mice vs. sex-matched WT mice……………………………………………………………………S6
Figure S3. Comparison of gene expression of glial fibrillary acidic protein (Gfap), allograft inflammatory factor 1 (Aif1), and an oxidative stress marker, sirtuin-3 (Sirt3), in the brain cortical tissue of female and male JNPL3 mice vs. sex-matched WT mice………………………………S7

Supplementary Table S1. *Characteristics of WT and JNPL3 mice. Individual abundances of investigated proteins (fmol/µg total protein) in the crude membrane fraction of the brain cortical tissue of female (F) and male (M) WT and JNPL3 mice (n = 8-9).*

# animal	Sex	WT / JNPL3	Body weight (g)	protein abundance in the brain cortex (fmol/ug total protein)								
				ABCB1ab	ASCT1	FATP1	4F2hc	GLUT1	ABCG2	ABCC1	LRP1	Na+/K+-ATPase
**1825414**	F	WT	34.8	0.266	5.31	0.775	1.06	9.58	0.780	0.667	1.19	313.0
**1825415**	F	WT	44.3	0.167	3.40	0.455	0.978	4.17	0.306	0.360	0.806	232.0
**1825416**	F	WT	38	0.289	2.05	0.495	0.983	5.77	0.859	0.538	1.05	205.0
**1825417**	F	WT	37.7	0.144	1.67	0.324	0.723	3.95	0.508	0.293	0.782	241.0
**1825418**	F	WT	34.3	0.302	3.32	0.599	0.795	4.71	0.480	0.342	0.707	249.0
**1825419**	F	WT	47.5	0.274	2.81	0.419	0.827	4.89	0.480	0.442	0.788	192.0
**1825420**	F	WT	38.2	0.174	1.73	0.512	0.827	5.17	0.330	0.327	0.636	280.0
**1825421**	F	WT	32.6	ULQ	1.67	0.252	0.480	2.08	0.287	0.182	0.386	123.0
**1825423**	M	WT	42.2	0.510	3.98	1.50	1.94	11.9	1.33	0.480	2.69	307.0
**1825424**	M	WT	44.5	0.251	3.39	0.934	1.11	8.69	0.424	0.360	1.42	252.0
**1825425**	M	WT	57.4	0.413	5.99	0.998	1.86	9.22	0.948	0.870	1.84	422.0
**1825426**	M	WT	40.1	0.332	5.00	0.451	1.43	5.02	0.326	0.420	0.909	257.0
**1825427**	M	WT	48.2	0.247	2.29	0.582	0.909	4.42	0.401	0.423	0.895	228.0
**1825428**	M	WT	55.2	ULQ	1.53	0.239	0.331	2.53	0.195	ULQ	0.338	142.0
**1825429**	M	WT	37.8	0.425	3.08	0.557	1.20	6.14	0.394	0.443	0.874	258.0
**1825430**	M	WT	35	0.383	2.84	0.601	0.795	5.03	0.263	0.456	0.976	232.0
**1825432**	M	WT	52.2	0.154	1.01	0.315	0.445	3.14	0.176	0.270	0.500	175.0
**1825252**	F	JNPL3	30.1	0.128	3.16	0.482	0.972	5.67	0.444	0.530	0.878	216.0
**1825253**	F	JNPL3	23.4	0.243	1.47	0.498	0.584	4.26	0.255	0.368	0.544	204.0
**1825254**	F	JNPL3	24.8	0.199	1.61	0.586	0.512	6.35	0.353	0.525	0.595	251.0
**1825255**	F	JNPL3	25.5	0.207	1.41	0.411	0.677	4.90	0.355	0.382	0.709	253.0
**1825256**	F	JNPL3	28.4	1.19	2.57	0.707	0.856	5.77	0.186	0.259	0.863	298.0
**1825257**	F	JNPL3	36.2	0.512	1.36	0.423	0.661	4.99	0.373	0.284	0.668	239.0
**1825258**	F	JNPL3	31.4	0.203	0.900	0.359	0.483	3.14	0.320	0.238	0.429	160.0
**1825259**	F	JNPL3	39.6	0.173	1.46	0.430	0.617	4.95	0.320	0.253	0.761	240.0
**1825260**	F	JNPL3	29.7	0.166	1.10	0.392	0.742	5.97	0.267	0.266	0.670	235.0
**1825276**	M	JNPL3	36.1	0.341	6.91	0.552	1.55	5.26	0.398	0.330	0.960	229.0
**1825277**	M	JNPL3	30.4	0.208	1.58	0.350	0.628	4.15	0.287	0.323	0.629	197.0
**1825278**	M	JNPL3	38.5	0.185	1.05	0.474	0.532	3.97	0.299	0.468	0.509	230.0
**1825279**	M	JNPL3	46.3	0.232	0.713	0.450	0.476	3.57	0.263	0.308	0.543	250.0
**1825280**	M	JNPL3	34.7	0.321	0.625	0.470	0.497	4.07	0.361	0.274	0.565	262.0
**1825281**	M	JNPL3	37.4	0.224	2.18	0.510	0.836	4.87	0.330	0.407	0.896	264.0
**1825282**	M	JNPL3	30.1	0.236	1.93	0.350	0.627	5.33	0.308	0.408	0.649	204.0
**1825283**	M	JNPL3	33.8	0.200	4.83	0.557	1.660	4.72	0.429	0.322	1.12	238.0
**1825284**	M	JNPL3	42.1	0.262	3.00	0.450	1.10	3.57	0.239	0.289	0.739	218.0

Supplementary Table S2. *Primer sequences for qRT-PCR analysis*

Gene	Forward primer	Reverse primer
Gfap	5′-CACCTACAGGAAATTGCTGGAGG-3′	5′-CCACGATGTTCCTCTTGAGGTG-3′
Aif1	5′-GGCTTCAAGTTTGGACGGCAGATCCTC-3′	5′-CATGAGCCAAAGCAGGGATTTGCAGGG-3′
Sirt3	5′-GCTACATGCACGGTCTGTCGAA-3′	5′-CAATGTCGGGTTTCACAACGCC-3′
Actb	5′-AAGTCCCTCACCCTCCCAAAAG-3′	5′-ACACAGAAGCAATGCTGTCACC-3′

Supplementary Table S3. *Probe peptide amino acid sequences and multiple reaction monitoring transitions for the LC-MS/MS-based quantitative targeted absolute proteomics analysis.*

**Protein/ gene name**	**St/ IS**	**Unique amino acid sequence**	**Retention time (min)**	**MRM transitions (m/z)**				
				**Q1**	**Q3.1**	**Q3.2**	**Q3.3**	**Q3.4**
			**ABC transporters**					
**ABCB1/Abcb1**[a]	St	NTTGALTTR	8.8	467.7	719.4	618.3	561.3	
	IS	NTTGALTT**R***	8.8	472.7	729.4	628.3	517.3	
**ABCG2/Abcg2**	St	SSLLDVLAAR	27.8	522.8	757.4	644.3	529.3	
	IS	SSLLDVLAA**R***	27.8	527.8	767.4	654.3	539.3	
**ABCC1/Abcc1**	St	TPSGNLVNR	10.1	479.2	759.4	672.3	501.3	
	IS	TPSGNLVN**R***	10.1	484.2	769.4	682.3	511.3	
			**SLC transporters**					
**ASCT1/ Slc1a4**	St	ETVDSFLDLLR	32.7	654.3	978.5	863.5	776.5	
	IS	ETVDSFLDLL**R***	32.7	659.3	988.5	873.5	786.5	
**GLUT1/Slc2a1**	St	TFDEIASGFR	21.3	571.7	894.4	779.4	650.4	537.3
	IS	TFDEIASGF**R***	21.3	576.7	904.4	789.4	660.4	547.3
**4F2hc/Slc3a2**	St	VAGSPGWVR	14.8	464.7	829.4	758.4	701.4	
	IS	VAGSPGWV**R***	14.8	469.7	839.4	768.4	711.4	
**FATP1/Slc27a1**	St	LLPQVDTTGTFK	20.7	660.4	1093.6	996.5	868.4	769.4
	IS	LLPQVDTTGTF**K***	20.7	664.4	1101.6	1004.5	876.4	777.4
			**Receptors**					
**LRP1/Lrp1**	St	GDYSVLVPGLR	24.1	588.3	840.5	753.5	654.4	442.2
	IS	GDYSVLVPGL**R***	24.1	593.3	850.5	763.5	664.4	452.2
			**Plasma membrane marker, abluminal membrane**					
**Na^+/K^+– ATPase**	St	AAVPDAVGK	10.9	414.3	685.4	586.3	489.3	
	IS	AAVPDAVG**K***	10.9	418.3	693.4	594.3	497.3	

[a]ABCB1 refers to both ABCB1a and ABCB1b
St – standard, IS – internal standard
Bold letter with* denotes labelled arginine (R) or lysine (K) with a stable isotope ^{13}C and ^{15}N

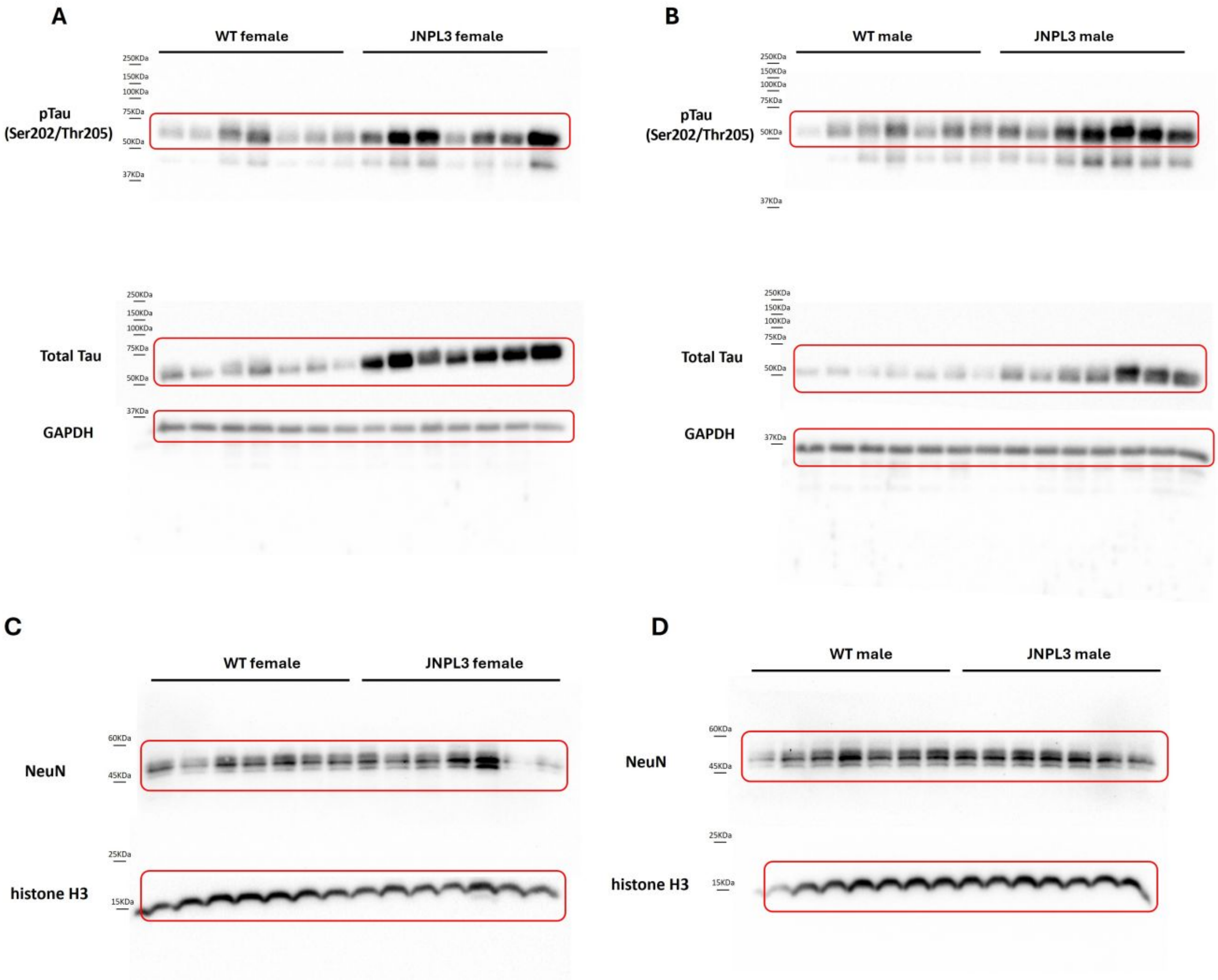

Supplementary Figure S1. *Original Western blots of p-tau and Total tau protein abundance in brain cortical samples from the whole homogenates of female JNPL3 mice (A; n = 7) and male JNPL3 mice (B; n = 7) compared to age-matched WT. Original Western blots of NeuN abundance in nuclear fractions isolated from the brain cortex of female JNPL3 mice (C; n = 7) and male JNPL3 mice (D; n = 7) compared with age-matched WT mice. Red boxes indicate which part of the unmodified image was used in the figure. GAPDH and histone H3 were used as housekeeping controls for whole homogenates and nuclear fractions, respectively.*

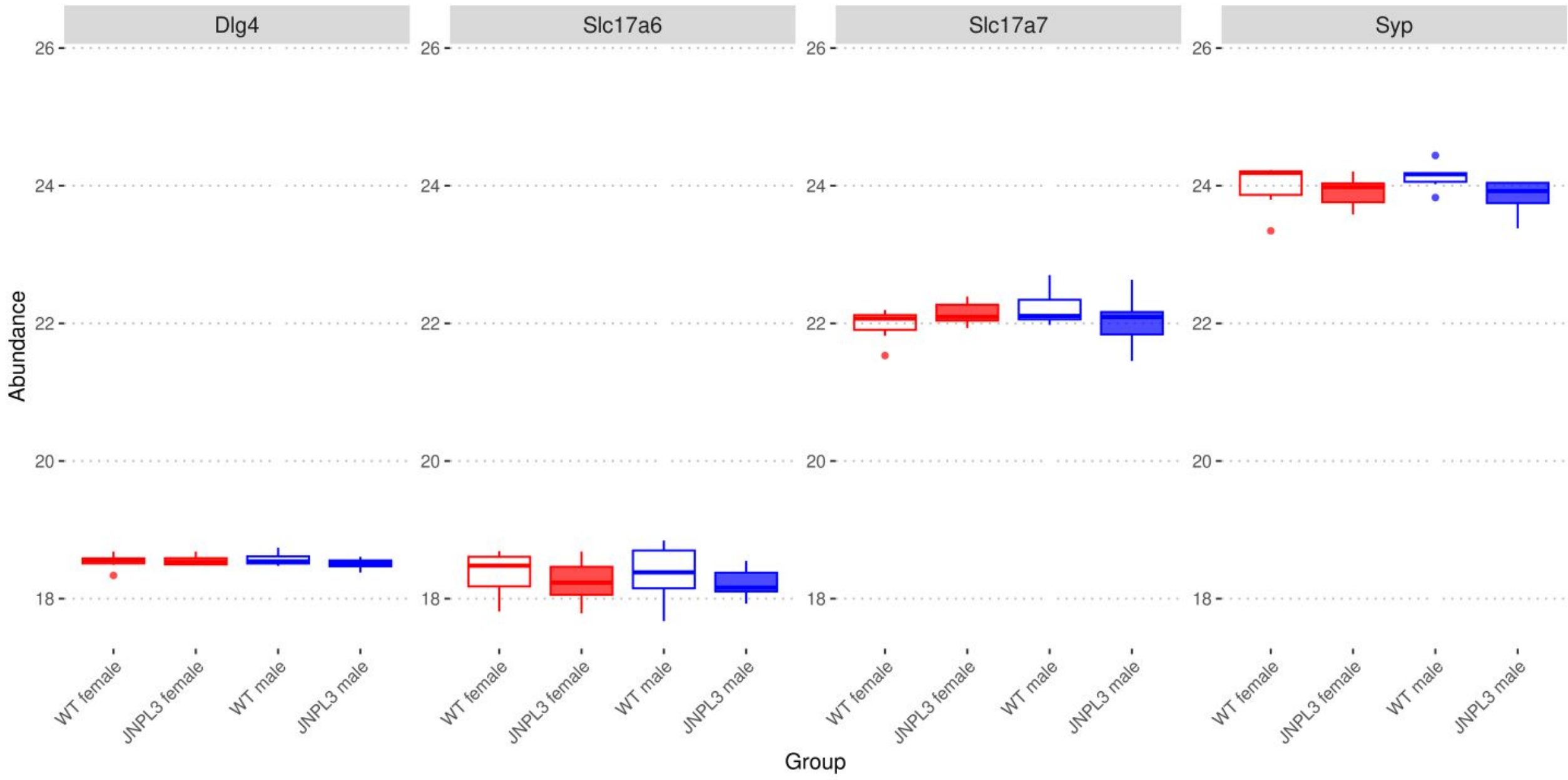

Supplementary Figure S2. *Normalised and transformed abundances of neuronal membrane proteins, including mouse Dlg4, Slc17a6, Slc17a7, Syp, presented as boxplots, grouped by sex and genotype. Data for female mice are coloured red, and for male mice are coloured blue. Filled boxes represent the JNPL3 mice, while empty boxes represent the WT mice. Brackets above the plots indicate statistically significant differences, defined as an adjusted p-value < 0.05 and an absolute log2 fold change (logFC) greater than 1.*

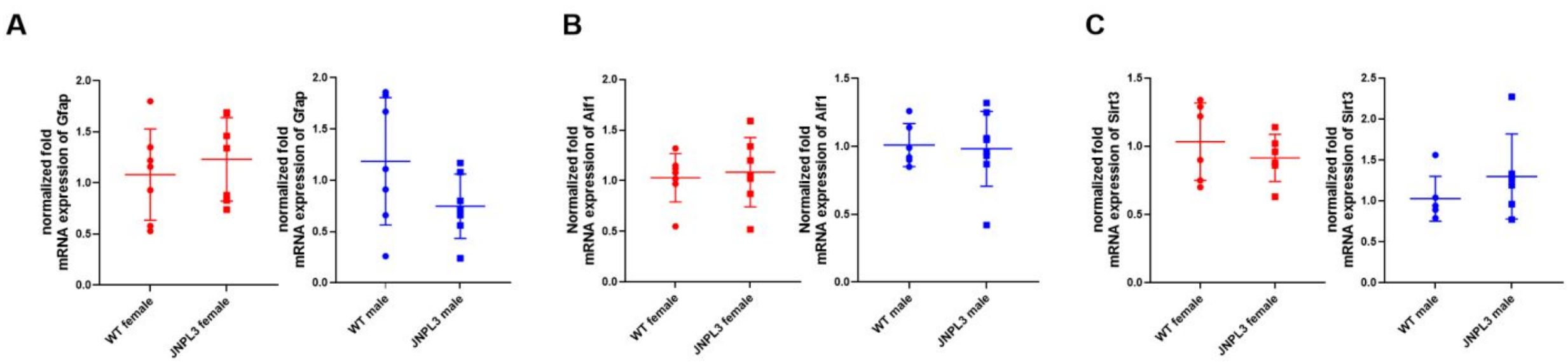

Supplementary Figure S3. *Comparison of gene expression of glial fibrillary acidic protein (Gfap)(A), allograft inflammatory factor 1 (Aif1)(B) and an oxidative stress marker sirtuin-3 (Sirt3) (C) in the brain cortical tissue of female and male JNPL3 mice vs. sex-matched WT mice (n = 6-7 per group). The gene expression was normalised against the housekeeping gene, beta-actin (Actb). The data are presented as mean ± SD. Statistical significance of differences between JNPL3 mice vs. corresponding sex-matched WT controls was analysed using an unpaired t-test.*
